# Dental Bibliography

**Published:** 1885-11

**Authors:** 


DENTAL BIBLIOGRAPHY.—A standard reference list of books on dentistry published throughout the world from 1536 to 1885. Arranged chronologically and supplemented with a complete cross-reference to authors. Compiled by C. Geo. Crowley. Publishers: The S. S. White Dental Manufacturing Co., Philadelphia. 1885. Price, cloth, $2.00.

This work of 180 pages is a valuable addition to dental literature, and must prove of great service to the practitioner who is interested in the literature of dental subjects. It is the first attempt to furnish information, which heretofore could only be obtained with great difficulty and after long and patient research.

The compiler is certainly worthy of the thanks of the profession for such a valuable list, the collection of which entailed great labor. The publishers also should receive the credit of placing such a collection in the hands of the intelligent dentist, and for enabling the compiler to present his work in the handsome style in which it appears. The work is divided into five departments or sections. Section I contains books published in Germany, Austria, Holland, Norway, Sweden, Denmark, and Switzerland (German); Section II books published in France, Belgium, and Switzerland (French); Section III books published in Spain and Italy; Section IV books published in Great Britain and Ireland; Section V books published in America. An author's index appended in alphabetical order gives cross-reference to all the volumes catalogu d. As a labor of love and not of profit, this publication should receive the high apprec ation it deserves.

